# E3-ligase Skp2 predicts poor prognosis and maintains cancer stem cell pool in nasopharyngeal carcinoma

**DOI:** 10.18632/oncotarget.2149

**Published:** 2014-06-30

**Authors:** Jing Wang, Ying Huang, Zhong Guan, Jia-liang Zhang, Hong-kai Su, Wei Zhang, Cai-feng Yue, Min Yan, Su Guan, Quentin Qiang Liu

**Affiliations:** ^1^ State key laboratory of oncology in South China, Collaborative Innovation Center of Cancer Medicine, Department of Research Laboratory, Sun Yat-sen University Cancer Center, Guangzhou, China; ^2^ Department of Research Laboratory, Sun Yat-sen University Cancer Center, Guangzhou, China; ^3^ Department of Otorhinolaryngology, Sun Yat-sen Memorial Hospital, Sun Yat-sen University, Guangzhou, China; ^4^ School of Bioscience and Biotechnology, South China University of Technology, Guangzhou, China

**Keywords:** S-phase kinase associated protein 2 (Skp2), nasopharyngeal carcinoma (NPC), aldehyde dehydrogenase1 (ALDH1), cancer stem cell (CSC)

## Abstract

Nasopharyngeal carcinoma (NPC) is one of the severe head and neck carcinomas, which is rare in west countries but has high incidence in Southern Asia especially South China. Although NPC is relatively sensitive to radiotherapy, the prognosis of patients is poor due to the advanced stage at the time of diagnosis. Therefore, it is important to understand the mechanisms involved in tumorigenesis and develop early diagnostic techniques. S-phase kinase associated protein 2 (Skp2) is overexpressed in several human cancers and associates with poor prognosis. However, its function in NPC has not been fully addressed. In this study we found Skp2 was highly expressed in NPC specimen and correlated with poor prognosis. We generated *Skp2* knockdown cells to further delineate its role in NPC development. Knockdown of *Skp2* partially reduced cell proliferation, promoted cellular senescence, and decreased the population of stem cell like aldehyde dehydrogenase1 positive cells as well as their self-renewal ability. Our study not only interprets the predictive role of Skp2 in the poor prognosis of NPC patients, but also reveals that Skp2 regulates the NPC cancer stem cell maintenance, which shed lights on the target therapy and early diagnosis of NPC in clinical application.

## INTRODUCTION

Nasopharyngeal carcinoma (NPC) is one of the most common head and neck cancers in Southern Asia and Northern Africa, the incidence reaches 25 per 100,000 people which is only 0.5~2 per 100,000 people in European and American [1]. The major etiologic factors for NPC are genetic susceptibility, endemic environment factors, and Epstein-Barr Viral infection. Early diagnosis is important for NPC patients since it is sensitive to radiotherapy at the early stage. However, about 30% to 40% of NPC patients were diagnosed at advanced stage, which are not response well to treatments and will develop metastasis or recurrence at an average of 4 years.

S-phase kinase-associated protein 2 (Skp2), a member of F-box protein family, is the substrate recognition subunit of Skp1-Cullin-F box protein (SCF) E3 ubiquitin ligase complex [2]. Early reports demonstrated that Skp2 recognizes and targets cell cycle inhibitors p21^Cip1/WAF^ and p27^Kip1^, leads to their ubiquitination and degradation, and further causes cell cycle progression [3]. Skp2 is overexpressed and associated with poor prognosis in variety of human cancers, including prostate cancer [4], gastric cancer[5], breast cancer [6, 7], and liver cancer [8], suggesting the oncogenic role of Skp2 in tumorigenesis. However, the association between Skp2 and NPC development and prognosis still remains unclear [9].

Cancer stem cells (CSCs) have been considered as the origin of tumorigenesis, therapeutic resistance, recurrence and distant metastasis [10-12]. The CSC like side population cells in NPC were first defied by our group with Hoechst 33342 staining [10]. Another functional assay for CSC identification is aldehyde dehydrogenase 1 (ALDH1) staining. Wu et al. firstly found that ALDH1 positive NPC cells express high level of stem cell related proteins like OCT4, Bmi-1, KLF4 and Sox2. These cells have high proliferation rate and differentiation ability, strong colony/sphere formation and migration ability compared with negative counterparts. Multivariate analysis indicates that ALDH1 could be an independent prognostic marker for NPC [13, 14]. Cell surface marker staining is another reliable method for CSC isolation. CD44 is a cell surface proteoglycan and glycoprotein related with body immune reaction [15, 16]. Stem cell markers OCT4 and Bmi-1 were highly expressed in CD44 positive NPC cells which are resistant to radiotherapy and cisplatin/docetaxel treatment [17]. CD133, another cell surface glycolprotein, is originally reported as the specific marker of normal stem cells. CD133 positive NPC cells exhibited strong self-renewal, proliferation and differentiation ability, as well as the remarkable tumor formation ability *in vivo* [18].

In this study, we demonstrate that Skp2 is a potential poor prognosis marker for NPC patients, inactivation of Skp2 decreases the NPC CSC population as well as their self-renewal ability. Our finding not only strengthens the role of Skp2 in the tumorigenesis of NPC but also indicates a potential target for NPC therapy.

## RESULTS

### High level of Skp2 relates with recurrence and metastasis among NPC clinicophathologic features

**Figure 1:**
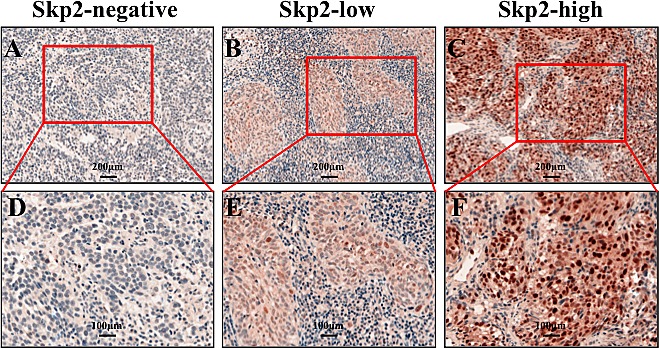
Skp2 expression levels in NPC specimen A, B and C: Representative images of negative, low and high staining of Skp2, which showed no, few and much brown-yellowish granules in the nuclei of tumor cells (×200). D, E and F: The magnification of the indicated field of A, B and C to show the clear staining granules (×400).

**Figure 2:**
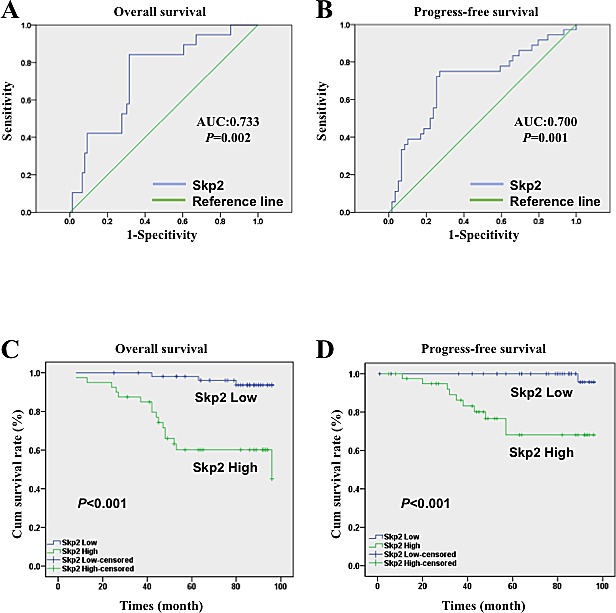
ROC curve and survival analysis of NPC patients with different level of Skp2 A, B: The ROC curve of Skp2 expression level in NPC patients (n=95), the area under curve was 0.733 and 0.700, the cutoff value was 131.25 and 128.82 for OS and PFS respectively (AUC: area under the curve). C, D: High level of Skp2 was closely related with poor OS (p<0.001) and PFS (p<0.001) of NPC patients (n=95).

IHC was employed to evaluate Skp2 expression levels in NPC specimens. The immunoreactivity of Skp2 was negative in normal tissue but increased in tumor tissues, where was stained as yellowish brown granules in the nuclei (Fig 1A-F). The signals were collected by microscope and analyzed by Nuance VIS-FL Multispectral Imaging System. We first performed ROC curve analysis (Fig 2A-B, the blue lines indicated the curve of Skp2, the green lines represents the curve of a completely indiscriminate). The cutoff points of OS and PFS from ROC curve analysis were 131.25 and 128.82 respectively. The areas under curve (AUC) were 0.733 and 0.700 for OS and PFS, and both of them were higher than 0.5 (Fig 2A-B). Skp2 high expression was examined in 42.1% (40/95) and low expression was examined in 57.9% patients (55/95). The association between Skp2 level and clinical features of patients, including age, gender, histopathologic characteristics, lymph node status, initial clinical stage, tumor stage, recurrence and metastasis were summarized in Table 1. High level of Skp2 was positively correlated with recurrence (p=0.005) and metastasis (p=0.037). Furthermore, the recurrent rate in patients with high Skp2 was higher in the first 3 years than later on follow-up time (12.5% versus 7.5%, Table 2).

**Table 1:** Associations between Skp2 level and clinicopathologic characteristics in NPC patients (n = 95)

Variable	All cases	Skp2		*p-value*
		High	Low	
Age				
< 44	51	21	30	0.844
≥ 44	44	19	25	
Gender				
Male	69	29	40	0.980
Female	26	11	15	
Histopathologic characteristics				
Undifferentiated carcinoma	93	40	53	0.223
Others	2	0	2	
Lymph node status				
Positive	17	5	12	0.242
Negative	78	35	43	
Initial clinical stage				
I+II	26	10	16	0.659
III+IV	69	30	39	
Tumor stage				
T_1_+T_2_	34	13	21	0.769
T_3_+T_4_	61	27	34	
Recurrence				
Yes	11	9	2	0.005
No	84	31	53	
Metastatic				
Yes	17	11	6	0.037
No	78	29	49	

**Table 2:** Estimated recurrence rate before and after 3 years in our NPC cohort

	Recurrent No. before 3 years			Recurrent No. after 3 years		
Skp2	%	*p-value*	%	*p-value*
Low (55)	0/55	0.00%	0.007	2/55	3.64%	0.410
High (40)	5/40	12.50%		3/40	7.50%	
Total (95)	5/95	5.26%		5/95	5.26%	

### High level of Skp2 indicates poor prognosis of NPC patients

With the cutoff point determined by ROC curve analysis, we separated Skp2 expression into high group and low group. After the Kaplan-Meier survival analysis, we found that high level of Skp2 was a strong indicator for an inferior OS (p<0.001, Fig 2C) and PFS (p<0.001, Fig 2D) in our patient cohort. The survival rate of patients with high and low levels of Skp2 were 60% (24/40) versus 94.5% (52/55) for OS, and 75.6% (31/41) versus 98.1% (53/54) for PFS respectively. In univariate analysis, high level of Skp2 predicted the poor OS (p<0.001, Hazard Ratio 10.122, 95% CI 2.931 to 34.952, Table 3) and PFS (p<0.001 Hazard Ratio 5.16, 95% CI from 2.472 to 10.771, Table 4) respectively. In multivariate analysis, Skp2 was also a significant predictor for poor OS (p=0.002, Hazard Ratio 8.215, 95% CI from 2.236 to 30.172, Table 3) and poor PFS (p=0.005, Hazard Ratio 3.488, 95% CI from 1.463 to 8.317 Table 4). Furthermore, recurrence and metastatic were also independent prognostic factors for PFS no matter from univariate or multivariate analysis (p<0.001, Table 4).

**Table 3:** Univariate and multivariate Cox-regression analysis for overall survival in NPC patients (n = 95)

Variable	Univariate analysis (OS)			Multivariate analysis (OS)		
	Hazard Ratio	95% confidence interval	*p*	Hazard Ratio	95% confidence interval	*P*
Age ≥44 (VS. <44)	1.119	0.454 to 2.756	0.807	1.177	0.438 to 3.160	0.747
Gender Male vs. Female	0.930	0.334 to 2.587	0.889	1.265	0.425 to 3.763	0.672
Histopathologic characteristics Undifferentiated carcinoma (VS. others)	0.048	0 to 1.636E4	0.640	0.000	0.000	0.987
Lymph node status Positive (VS. Negative)	4.800	0.640 to 36.034	0.127	2.531	0.319 to 20.061	0.379
Initial clinical stage III+IV (VS. I+II)	2.284	0.665 to 7.847	0.190	2.226	0.338 to 14.667	0.406
Tumor stage T_3_+T_4_ (VS. T_1_+T_2_)	1.785	0.642 to 4.963	0.266	1.249	0.253 to 6.179	0.785
Recurrence Yes (VS. No)	2.553	0.842 to 7.741	0.098	1.143	0.353 to 3.700	0.824
Metastatic Yes (VS. No)	3.538	1.386 to 9.031	0.008	3.021	1.038 to 8.794	0.043
Skp2 High (Vs. No)	10.122	2.931 to 34.952	<0.001	8.215	2.236 to 30.172	0.002

**Table 4:** Univariate and multivariate Cox-regression analysis for progression-free survival in NPC patients (n = 95)

Variable	Univariate analysis (PFS)			Multivariate analysis (PFS)		
	Hazard Ratio	95% confidence interval	*P*	Hazard Ratio	95% confidence interval	*P*
Age ≥44 (VS. <44)	1.334	0.693 to 2.568	0.389	1.630	0.758 to 3.506	0.211
Gender Male vs. Female	0.869	0.408 to 1.852	0.716	1.541	0.686 to 3.463	0.295
Histopathologic characteristics Undifferentiated carcinoma (VS. others)	2.431	0.331 to 17.875	0.383	10.591	1.155 to 97.078	0.037
Lymph node status Positive (VS. Negative)	10.460	1.430 to 76.513	0.027	3.730	0.485 to 25.684	0.206
Initial clinical stage III+IV (VS. I+II)	1.915	0.836 to 4.388	0.124	1.276	0.376 to 4.325	0.696
Tumor stage T_3_+T_4_ (VS. T_1_+T_2_)	1.402	0.698 to 2.816	0.342	1.433	0.495 to 4.147	0.507
Recurrence Yes (VS. No)	7.138	3.370 to 15.120	<0.001	6.000	2.541 to 14.165	<0.001
Metastatic Yes (VS. No)	6.959	3.579 to 13.532	<0.001	8.561	3.773 to 19.427	<0.001
Skp2 High (Vs. No)	5.160	2.472 to 10.771	<0.001	3.488	1.463 to 8.317	0.005

### Skp2 is high expressed in poor differentiated NPC cell lines

**Figure 3:**
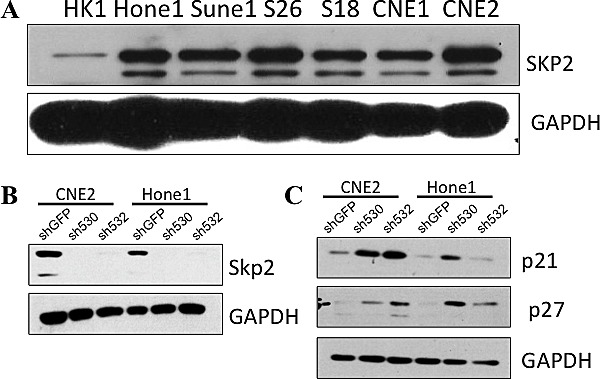
Skp2 expresses high in several NPC cell lines A: Skp2 was highly expressed in poor-differentiated cell lines (Hone1, Sune1, S26, S18 and CNE2), but relatively low in well-differentiated cell lines (HK1 and CNE1). B: *Skp2* was successfully knocked down in CNE2 and Hone1 cells compared with vector control. C: Both of p21^Cip/WAF^ and p27^Kip^ were upregulated in CNE2 and Hone1 cells upon *Skp2* knockdown.

Since Skp2 was a poor prognostic factor for NPC patients, we next sought to define its level in cellular level (Fig 3A). We found that Skp2 was relatively higher in poor-differentiated cell lines, including CNE2, Hone1, Sune1 as well as two other cell lines derived from CNE2, S26 and S18. While in well-differentiated cell lines (HK1 and CNE1), Skp2 expression was low especially in HK1 cells. We chose CNE2 and Hone1 for following experiments since most of the NPC are poor-differentiated pathologically. To further study the role of Skp2 in NPC progression, we knocked down Skp2 with two specific short hairpin RNAs (shRNAs) in CNE2 and Hone1 cells. The expression level of Skp2 was significantly reduced in both CNE2 and Hone1 cells compared with shGFP control (Fig 3B). In order to exclude off-target effect, we detected well-known downstream targets of Skp2, p21^Cip/WAF^ and p27^Kip^, and found both of their levels were elevated upon *Skp2* knocking down in CNE2 and Hone1 cells (Fig 3C).

### Skp2 inactivation partially reduces cell proliferation and triggers cellular senescence

**Figure 4:**
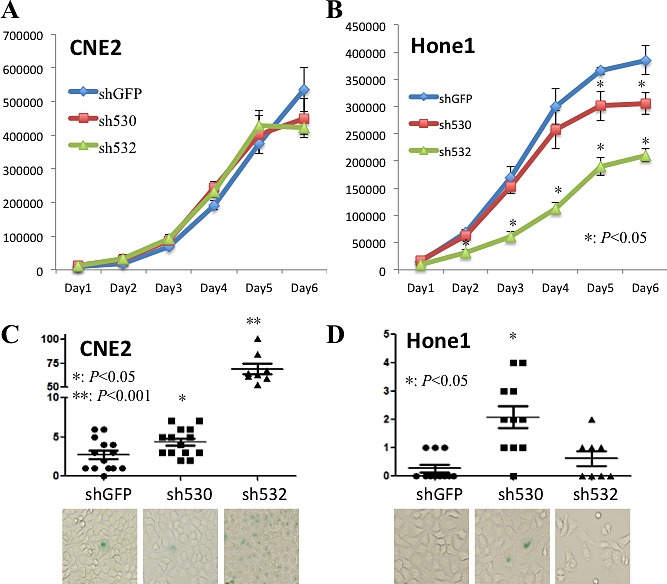
Skp2 deficiency partially reduces cell proliferation and triggers cell senescence A, B: The cell proliferation rate did not change significantly upon *Skp2* knockdown in CNE2 cells, whereas decreased dramatically by both of the fragments in Hone1 cells, starting from the fifth and second day respectively (*: p<0.05). C, D: Cellular senescence was enhanced by both of the knockdown fragments in CNE2 cells and only one of the fragments in Hone1 cells (*: p<0.05, **p<0.001), lower panels showed the cell images under bright field.

We next determined the role of Skp2 in NPC cell growth since it's a known cell cycle regulator. There was no significant change in the cell cycle profile of CNE2 and Hone1 cells after *Skp2* knockdown (data not shown). However, inconsistent effects were found on CNE2 and Hone1 cells. For the cell proliferation study, there was no growth retardation after *Skp2* knockdown in CNE2 cell (Fig 4A). But in Hone1 cells, both of the knockdown fragments attenuated cell proliferation compared with control cells, the effect started from day 2 and day 5 respectively (p<0.05, Fig 4B). Moreover, it has been reported that *Skp2* inactivation promotes the senescence of prostate cancer cells [19]. We then detected cell senescence and interestingly found that knockdown of *Skp2* increased SA-βGal positive cells in both CNE2 and Hone1 cells: from 2.54 ± 1.98 per field to 4.36± 1.74 (p<0.05) and 68.88 ±15.89 (p<0.001) in CNE2 cells (Fig 4C), from 0.27±0.47 per field to 3.12 ±2.85 (p<0.05) in Hone1 cells (one of fragment had no effect, p>0.05, 0.63 ± 0.22) (Fig 4D). Enhanced cellular senescence was also found in well-differentiated NPC cell line CNE1 without obvious cell proliferation retardation (Supplementary Fig S1). These findings indicated that although Skp2 does not play a universal role in all NPC cells lines, but indeed involves in cell proliferation and senescence.

### Knockdown *Skp2* decreases CSC population and self-renewal ability

**Figure 5:**
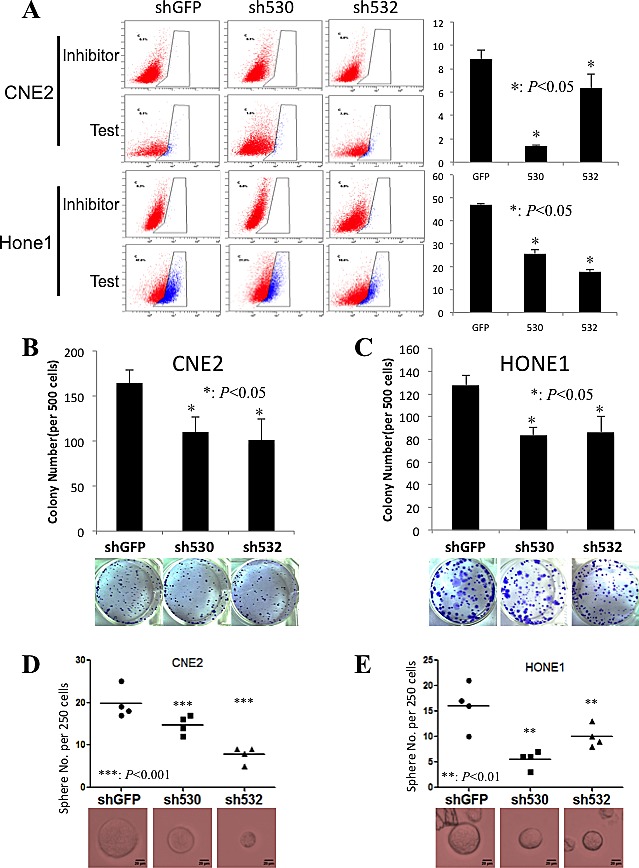
CSC population and the self-renewal ability decrease upon *Skp2* knockdown A: The ALDH1 positive stem cell frequency dropped dramatically in both CNE2 and Hone1 cells by two knockdown fragments comparing to control cells (*: p<0.05), left panel presents the flow cytometry profile and right panel displays the statistical results. B, C: The colony formation ability of both CNE2 and Hone1 cells decreased in Skp2 deficient cells (*: p<0.05), lower panels were the representative images of wells with colonies. D, E: The sphere formation ability was reduced significantly in both CNE2 (from19.76±3.6 to 14.75±2.22 and 7.75±1.89) and Hone1 (from 12.25±1.71 to 5.25±1.71 and 4.75±1.71) cells upon *Skp2* knockdown (***: p<0.001, **: p<0.01)

Our previous report showed that *Skp2* inactivation leads to the reduction of ALDH1 positive cell frequency and involves in the breast CSC maintenance[20]. We therefore detected ALDH1 positive NPC cells after *Skp2* knockdown. As expected, ALDH1 positive cells frequency were reduced from 8.8±0.75% to 1.4±0.1% (p<0.001) and 6.3±1.21% (p<0.05) in CNE2, and from 46.93±0.76% to 25.83% (p<0.001) and 17.8±0.92% (p<0.001) in Hone1 upon *Skp2* inactivation (Fig 5A). As an important characteristic of CSCs, self-renewal ability of cells was evaluated. The colony numbers from 500 cells on regular plate reduced from 166.67±14.5 to 110.33±16.5 and 101.0±23.26 in CNE2 (Fig 5B, p<0.05), while from 127.33±9.07 to 83.33±6.80 and 86.33±14.22 in Hone1 upon *Skp2* inactivation (Fig 5C, p<0.05). Furthermore, the sphere formation ability on low-attached plates was decrease dramatically from 19.76±3.6 to 14.75±2.22 and 7.75±1.89 in CNE2 (Fig 5D, p<0.001), from 12.25±1.71 to 5.25±1.71 and 4.75±1.71 in Hone1 (Fig 5E, p<0.01) and CNE1 (Supplementary Fig S2A). At the meanwhile, the anchorage independent colony formation ability was reduced significantly in CNE2 (51.00±3.61 to 29.00±4.58 and 22.33±6.03, p<0.01) (Supplementary Fig S2B). These data demonstrated that Skp2 involves in the maintenance of both pool size and self-renewal capacity of CSCs in NPC cell lines.

## DISCUSSION

Skp2 is an important cell cycle regulator, which is widely studied in various cancer types. However, the role of Skp2 in NPC was seldom reported. ROC curve analysis is widely used clinically to evaluate sensitivity and specificity of diagnostic tests, the selected threshold (cut-off value) is useful for clinicians to make decision according to laboratory tests. The method has been proved to be reliable in tumor marker comparison [21]. We performed ROC curve analysis to establish a cut-off point in order to help the clinical diagnosis and treatment. In our patient cohort, Skp2 was an independent poor prognosis indicator for both OS and PFS. Furthermore, Skp2 predicted high recurrence and migration risk at the early stage of patients. Our result is consistent with previous reports that indicate the poor predictive role of Skp2 in NPC patients [22, 23].

As a cell cycle modulator, *Skp2* deficiency is reported to slow down the cell proliferation in breast cancer, prostate cancer cells. However, there is no dramatic cell cycle arrest upon *Skp2* knockdown in NPC cell line CNE2 and Hone1. Literatures show that the oncogenic role of Skp2 always relies on the genetic status of PTEN, ARF, and Rb and other tumor suppression genes [19, 24]. However, the aberrant PTEN, P53 and RB are not frequent in NPC [25], which might lead to the inconsistent outcome upon *Skp2* knockdown accompanied by reduction of p21^cip/WAF^ and p27^Kip^. Further experiments are needed to delineate the role of Skp2 in the cell cycle regulation in NPC cells.

Cellular senescence is the status of permanent cell proliferation arrest which could be induced by eroded chromosome during repeated cell division and oncogenic stress [26]. Development of cellular senescence mostly depends on two fundamental tumor suppressor pathways ARF-p53 and p16-pRb. As our previous publication proved that *Skp2* silencing cooperated with PTEN inactivation and triggered cellular senescence by a p19^Arf^-p53 independent pathway. Furthermore, levels of p21, p27 and Atf4 were induced after the inactivation of *Skp2*. Knockdown of p21, p27 and Atf4 reversed cellular senescence consistently, which confirmed that the upregulation of p21 and p27 were required for the induction of cellular senescence upon *Skp2* knockdown [19]. We provided evidences in the current study that *Skp2* inactivation promoted the cellular senescence in NPC cell lines through p21^cip/WAF^ and p27^Kip^.

Cancer mass would relapse after most of non-CSC were killed by chemotherapy or radiotherapy due to the small amount of quiescent, high proliferate multipotent CSCs. In this study we found that knockdown *Skp2* led to the reduction of stem cell like ALDH1 positive NPC cells. At the mean while, the self-renewal ability was decreased in *Skp2* deficient cells. Previous we show that Skp2 plays an important role in the maintenance of quiescence and self-renewal ability of hematopoietic stem cell[27]. Moreover, we find that β-catenin is downregulated in hematopoietic stem cells from *Skp2* knockout mouse, which subsequently attenuates the homing ability of hematopoietic stem cell [28]. Here we propose that β-catenin is the downstream executor of Skp2 to modulate the CSC in NPC cells. In deed the role of β-catenin in NPC CSC has been reported recently. The stem cell population increases in NPC cell line Hone1 upon enforced expression of β-catenin[29]. Another report shows that β-catenin is the downstream executor of EGFR/AKT regulated CSC property reservation in NPC cells[30]. Our future work will focus on deciphering the connection between β-catenin and Skp2 in the background of NPC.

Since Skp2 not only involves in the prognosis of NPC but also regulates the stem cell pool size and biological function of NPC cell line, we propose that Skp2 could be a potential target for NPC therapy. CSC target therapy in NPC has been shown promising outcome. Phenolic compounds resveratrol suppresses stemness of NPC CSCs[31], and the epithelial mesenchymal transition (EMT) which is considered as one of the origin of CSCs[32]. Nigericin, an antibiotic derived from Streptomyces hygroscopicus targets NPC CSC both in vitro and in vivo [33]. Recently, a specific Skp2 inhibitor is identified using high-throughput screening from large and diverse chemical libraries. This inhibitor selectively suppresses Skp2 E3 ligase activity, exhibits antitumor function in multiple animal models and reduces cancer survival combining with other chemotherapeutics[20]. Taken together, targeting Skp2 could benefit the patients by suppression of both CSCs and non-CSCs.

Our study provided evidences that Skp2 could serve as a poor prognosis marker not only for overall survival but also for recurrence and metastasis of NPC patients. We also emphasized the important role of Skp2 in NPC stem cell population and self-renewal capacity maintenance. Our finding might facilitate clinician in the future diagnosis and treatment of NPC patient.

## MATERIAL AND METHODS

### Patients and cell lines

NPC specimens were obtained from Departments of Nasopharynx undergoing nasal endoscopy at Sun Yat-sen University Cancer Center (SYSUCC) with written informed consent (n = 95). Patients were diagnosed and classified by the Department of Pathology of SYSUCC following WHO guidelines. Age of patients ranged from 16 to 70, averaged 43.8 years old. Human nasopharyngeal carcinoma cell lines CNE1, CNE2, S18, S26, Hone1, Sune1 and HK1 were from State Key Laboratory of Oncology in South China. CNE1, CNE2, S18, and S26 were maintained in RPMI1640 (Gibco) with 10% fetal bovine serum (FBS, Gibco), Hone1, Sune1 and HK1 in RPMI1640 (Gibco) containing 10% FBS (Hyclone). All cells were cultured in humidified incubator at 37°C, 5% CO_2_. Investigation has been conducted in accordance with the ethical standards and according to the Declaration of Helsinki and according to national and international guidelines and has been approved by the SYSUCC institutional review board.

### Follow-up

All patients included in this study had follow-up records for over 5 y. After the completion of therapy, patients were observed at 3 m intervals during the first 3 y and at 6 m intervals thereafter. The latest follow-up information was updated in January 2014. Overall survival (OS) was defined as the time from completion of therapy to the date of death or when censored at the latest date if patients were still alive; progression-free survival (PFS) was defined as the time from completion of therapy to the date of disease relapse/progression or the date of death or when censored at the latest date.

### Immunohistochemical (IHC) staining and evaluation

IHC staining was performed as described previously [34]. In brief, after deparaffinization, rehydration, antigen retrieval, and blocking, slides were incubated overnight at 4°C with monoclonal antibody against human Skp2^p45^ (1:200; Invitrogen, USA) in a moist chamber. Next day the slides were incubated with secondary antibodies at room temperature for 30 min. Then slides were stained with 3, 3-diaminobenzidine and hematoxylin separately. Negative controls were achieved by replacing Skp2 antibody with corresponding non-immune serum immunoglobulin. The slides were scanned with the Nuance VIS-FL Multispectral Imaging System (Cambridge Research Instrumentation, Woburn, MA) following the vender's instructions. The signals were evaluated with Nuance 3.0 software referring to previous reports[35, 36].

### Receiver operating characteristic (ROC) curve analysis

Being a reproducible method, ROC curve analysis has been applied to assess the tumor markers for diagnosis and prognosis prediction. Herein, we first used ROC curve analysis to select the cutoff point of Skp2 expression levels in our patient cohort for OS and PFS. In brief, the score localized closest to the point at both maximum sensitivity and specificity (0.0, 1.0) was selected as the cutoff score, leading to the greatest number of tumors which were correctly classified as having or not having the outcome. ROC curve analysis was facilitated by dichotomizing the features of patients’ outcome into survival (death VS. others (censored, alive or death from other causes) and progression (local failure or distant metastasis).

### Viral infection

293T cells were co-transfected with packaging plasmids p-Helper, p-Envelope and short hairpin RNA (shRNA) using Lipofectamine 2000 (Invitrogen, USA) to produce lentivirus particles according to manufacturer's instructions. Skp2 lentiviral shRNA sequences were reported before[37]. Supernatant from 293T cells were collected 48 hr after transfection and applied to target cell. CNE2 and Hone1 cells were then selected by 1.5 μg/mL puromycin two days after infection. Two weeks after selection, cell lysates were collected for Western Blot analysis to confirm the knockdown efficiency.

### Western blot assay

Cells were harvested and lysed by RIPA lysis buffer (1% NP-40, 1% sodium deoxycholate, 0.1% SDS, 150 mM NaCl and 10 mM Na_2_HPO_4_, pH7.2), supplemented with protease inhibitor cocktail (Roche, Mannheim, Germany). Immunoblotting was performed with standard protocols as previously described. The following antibodies were used:anti-Skp2^p45^ (1:2000, Invitrogen), anti-p21^Cip/WAF^(1:1000, CST), anti-p27^Kip^(1:1000, CST), anti-GAPDH (1:10000, Protein Tec Group).

### Cell cycle profiles and proliferation analyses

Cells were harvested, washed with pre-cold PBS, and fixed with ice cold 70% ethanol for at least 30 min. Cells were then stained with propidium iodide (50 mg/ml, Sigma) and 100 μL RNase (1.0 mg/mL; Roche) for 30 min at 37°C against light and then were measured by flow cytometry. Cell proliferation was evaluated by cell counting. We seeded same amount of cells into 24-well plate and counted for six continuous days monitoring the cell growth (each day quadruplicate).

### Senescence-associated β-galactosidase Staining

Cells were plated into 6-well plate at the concentration of 1×10^5^ per well. After being cultured for around 3 to 4 days, cells were stained with Senescence Activated β-galactosidase (SA-βGal) Staining Kit (Beyotime, Guangzhou, China) following manufacturer's instructions. Then the green cells were observed under microscopy.

### Aldehyde Dehydrogenase 1 (ALDH1) positive cell detection

The ALDH1 positive cell was detected using ALDEFLUOR assay kit (Stem Cell Technologies, Canada) following manufacturer's instruction. Briefly, cells were suspended in ALDEFLUOR assay buffer containing ALDH1 substrate and incubated 40 min at 37°C. The aliquot of each sample cells was treated with 50 mmol of specific inhibitor of ALDH1 (diethylaminobenzaldehyde) as negative control, according to which we set up gate. Then the positive cells were detected by a standard flow cytometer in the green fluorescence channel (520-540 nm). Propidium iodide was used before flow cytometer analysis to exclude dead cells.

### Colony formation assay

Colony formation assay was performed according to procedures reported previously[10]. Briefly, cells were typsinized, resuspended into single cell supernatant, and then seeded into regular 6-well plates with 500 cells per well. After being cultured for about 14 days, the colonies were fixed with methanol and stained with crystal violet. Colonies with over 50 cells were counted.

### Sphere formation assay

The sphere formation assay was followed with previous report[38]. Cells were seeded into ultralow attachment 24-well plate (corning), 250 cells per well. Cells were grown in a DMEM/F12 medium (Gibico), supplemented with B27 (Invitrogen), EGF (20 ng/ml), bFGF (20 ng/ ml, BD Biosciences), and heparin (0.5U/ml, Sigma). Fresh media were added (200ul/well) and the plates were gently shaked every other day. The spheres were counted after being cultured for 10 to 15 days.

### Soft agar colony formation assay

The soft agar colony formation assays were performed according to the previous report [39]. Briefly, we re-suspended cells in medium containing 0.6% agarose (1,000 cells/well) and seeded into 6-well plate which was coated with 0.8% agarose. Then 2ml of culture media were added on the top in order to maintain the moisture and nutrient. After being cultured for about 2 to 3 weeks, the colonies were counted.

### Statistical Analysis

The cutoff value of Skp2 signals for OS and PFS were assessed with ROC curve analysis. The relationships between Skp2 level and OS, PFS were evaluated with Kaplan-Meier survival analysis. The association between clinicopathologic factors and Skp2 level was evaluated using Chi-square test. The hazard ratios and 95% confidence intervals for patient outcome were estimated by univariate and multivariate Cox regression. The data from in vitro study were expressed as mean ± standard error. All p values quoted were two sided and p<0.05 was considered statistically significant. Statistical analysis was performed with SPSS 16.0 (SPSS, Inc, Chicago, IL).

## SUPPLEMENTARY MATERIAL AND FIGURES


