# Supplementary material for: E3-ligase Skp2 predicts poor prognosis and maintains cancer stem cell pool in nasopharyngeal carcinoma
Source: Oncotarget. 2014 Jun 30;5(14):5591–601. doi: 10.18632/oncotarget.2149 (PMC4170633; doi:10.18632/oncotarget.2149)
Supplement: Supplementary file 1 [file oncotarget-05-5591-s001.pdf]

E3-ligase Skp2 predicts poor prognosis and maintains cancer stem cell pool in nasopharyngeal carcinoma

Supplementary Material

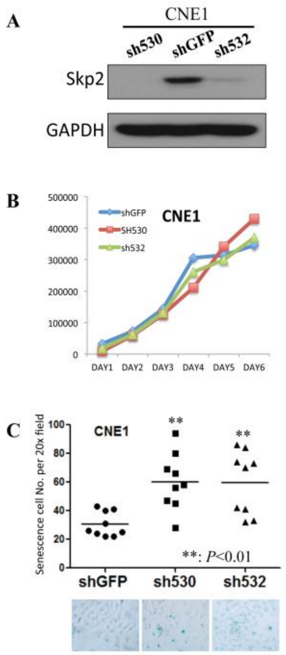

Supplementary figure legends Figure S1: Skp2 deficiency does not affect cell prolifera8on but triggers cell senescence in NPC cell line CNE1 A: The knockdown efficiency of Skp2 in CNE1 cell. Both of the two fragments worked well in CNE1. B: The cell proliferaBon rate did not change significantly upon Skp2 knockdown in CNE1 cell. C: Cellular senescence was enhanced by both of the knockdown fragments in CNE1 cell (**: $p<0.05$). Lower panels indicated the cell images under bright field.

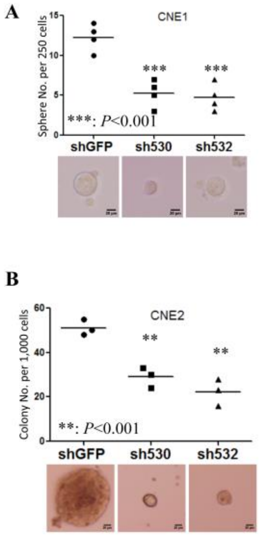

Figure S2: *Skp2* deficiency a@enuated the self---renewal ability of NPC cell lines A: The sphere formaBon ability was reduced significantly in *Skp2* deficient CNE1 cell (***: *p*<0.001). C, D: The soM agar colony formaBon ability reduced dramaBcally in *Skp2* deficient CNE2 cell (**: *p*<0.01).

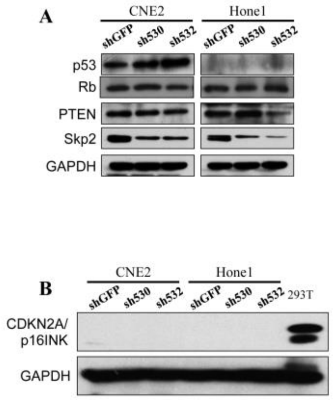

Figure S3: *Skp2* deficiency induced cellular senescence was not through PTEN, p19Arf/p53 or Rb/p16 pathways A: p53 was detected in CNE2 but not in Hone1. Levels of PTEN and Rb were comparable in *Skp2* silenced cells with control cells. B: p14(CDKN2A) and p16 was under detectable in CNE2 and Hone1 cells comparing to posiBve control HEK293T.
